# Risk factors for measles deaths among children during a Nationwide measles outbreak – Romania, 2016–2018

**DOI:** 10.1186/s12879-021-05966-3

**Published:** 2021-03-19

**Authors:** Morgane Donadel, Aurora Stanescu, Adriana Pistol, Brock Stewart, Cassandra Butu, Dragan Jankovic, Bogdan Paunescu, Laura Zimmerman

**Affiliations:** 1 Global Immunization Division, Centers for Disease Control and Prevention, 1600 Clifton Rd NE, Atlanta, GA 30333 USA; 2 National Public Health Institute, Bucharest, Romania; 3 World Health Organization, Romania Country Office, Bucharest, Romania; 4 World Health Organization, European Regional Office, Copenhagen, Denmark; 5 TOTEM Survey Company, Bucharest, Romania

**Keywords:** Measles, Mortality, Romania, Vitamin A supplementation, Nosocomial transmission, Vaccine

## Abstract

**Background:**

Case fatality ratio (CFR) among all age groups during the 2016–2018 measles outbreak in Romania was increased compared with previous outbreaks. To identify risk factors for measles death, we conducted a case-control study among infants and children hospitalized for measles.

**Methods:**

National surveillance data were used to identify hospitalized cases of laboratory-confirmed or epidemiologically linked measles in infants and children aged < 59 months with rash onset from January 2016 to July 2018. We abstracted medical records of 50 fatal cases (“cases”) and 250 non-fatal cases (“controls”) matched by age, sex, district of residence, and urban/rural place of residence. We calculated univariable and multivariable matched odds ratios (OR) and 95% confidence intervals (CIs) for risk factors.

**Results:**

Ninety-three percent of case-patients and controls had not received a valid dose of a measles-containing vaccine; only 5 % received Vitamin A supplementation once diagnosed with measles. In the univariable analysis, cases were more likely than controls to have had a healthcare-related exposure to measles manifesting as inpatient admission for pneumonia during the 7 to 21 day measles incubation period (OR: 3.0; 95% CI [1.2, 7.2]), to have had a history of malnutrition (OR: 3.4; 95% CI [1.1, 9.9]), and to have had pneumonia as a complication of measles (OR:7.1; 95% CI [2.0–24.8]). In the multivariable analysis, pneumonia as a measles complication remained a risk for death (OR: 7.1; 95% CI [1.4–35.3]).

**Conclusions:**

Implementing infection prevention and control practices, ensuring immunization of healthcare workers, and hospitalizing only severe measles cases may minimize the risk of nosocomial measles transmission. Implementing World Health Organization (WHO) recommendations for Vitamin A supplementation, improving immunization of children to prevent influenza, pneumococcal, and other bacterial respiratory diseases may decrease complications and deaths due to measles in Romania.

**Supplementary Information:**

The online version contains supplementary material available at 10.1186/s12879-021-05966-3.

## Background

Measles is a highly communicable viral disease transmitted from person-to-person by droplet or aerosols from the respiratory fluids of infected persons [1]. The incubation period for measles is 7–21 days. Symptoms of measles infection include fever, generalized maculopapular erythematous rash, cough, coryza, and conjunctivitis. Complications may occur up to several weeks after rash and include pneumonia (1:20 cases), otitis media (1:10 cases), thrombocytopenia, diarrhea (1:10 cases), and encephalitis (1:1000 cases). In malnourished children, measles may lead to blindness, deafness, intellectual disabilities associated with encephalitis (acute disseminated encephalomyelitis in approximately 1:1000 cases; subacute sclerosing panencephalitis in approximately 1:10,000 to 1:100,000 cases), and death (1─ 3:1000) [1]. Still responsible for 100,000 deaths annually, measles case fatality ratios range from less than one in 1000 cases to 5% in endemic areas in sub-Saharan Africa and Asia, to as high as 20–30% in refugees and internally displaced populations [1].

Romania, a middle-income country (MIC) in Europe, has recommended receipt of two doses of measles-containing vaccine (MCV) since 1994. Since 2002, the first dose (MCV1) is normally given at 12 months and the second dose (MCV2) at 5 years of age [2]. From 2000 through 2010, MCV1 coverage in Romania was > 95% annually. Starting in 2011, coverage with MCV1 began declining in Romania and was ≤86% annually during 2015–2017 [3]. A vocal and effective anti-vaccine lobby within the country and challenges within the immunization infrastructure related to the availability of vaccines for routine immunization and vaccine delivery contributed to this decline in national MCV coverage [4].

**Fig. 1:**
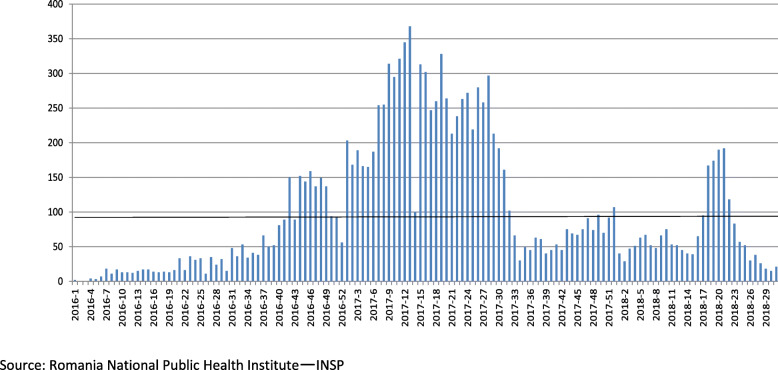
Reported epidemiologically-linked or laboratory-confirmed measles cases — Romania, January 2016–July 2018 (*N* = 14,825)**.** Source: Romania National Public Health Institute—INSP

Despite a World Health Organization (WHO) European Region goal of measles elimination, Romania continues to have large, nationwide measles outbreaks, including 8709 cases reported during 2004–2007 and 12,991 cases reported during 2010–2013 [5]. The most recent outbreak began in 2016, and as of 27 July 2018, the Romanian Ministry of Health (MOH) had reported 14,825 cases of measles. Of the 14,825 cases, 2435 (16.5%) were reported in 2016; 9076 (61.0%) were reported in 2017; and 3314 (22.5%) were reported through July 2018. The outbreak originally appeared to have peaked in May of 2017 (Fig. 1), however, a second peak was observed in May 2018. Of the 14,825 reported measles cases, 14,113 (95.2%) were unvaccinated, and 8259 (56%) of the cases were among infants and children under 5 years of age [6].

Fifty-nine measles deaths were reported during the outbreak, of which 50 (85%) were among infants and children under 5 years of age. The resultant measles case fatality ratio (CFR) was 0.4% compared with 0.2% during the 2004–2007 outbreak and 0.02% during the 2010–2013 outbreak [7]. Because of concern among healthcare providers and the public about the higher CFR in this outbreak, we conducted a case-control study to identify the risk factors associated with measles-related deaths among infants and children from January 2016 through July 2018.

## Methods

### Study population and vaccine dose

The case-control study population included hospitalized measles case-patients up to 59 months of age who had onset of illness from January 2016 through June 2018 in Romania and who were laboratory-confirmed or epidemiologically linked to a confirmed measles case. The laboratory criteria for measles surveillance case confirmation are measles Immunoglobulin M (IgM) antibody detection or measles virus isolation or measles viral ribonucleic acid (RNA) detection by reverse transcription polymerase chain reaction (RT-PCR) or a significant rise in measles Immunoglobulin G (IgG) antibody in paired sera. An epidemiologically linked case was defined as a case that had not been adequately tested at the laboratory and which had been in contact with a laboratory-confirmed measles case 7–21 days before the onset of rash. We defined a study case as a measles-related death among the hospitalized measles patients, based on the WHO definition — a death in an individual with a confirmed case of measles that occurs within 30 days of rash onset and is not due to another unrelated cause (e.g., trauma or chronic disease) [8]. We defined a study control as a non-fatal measles case, i.e., a hospitalized measles patient who did not die within 30 days of rash onset. We defined a valid dose of MCV as an MCV dose received > 9 months of age and > 28 days before rash onset.

### Sample size

In the absence of a known correlation coefficient between the exposure status of the i^th^ pair of the case and controls, Dupont (1988) suggests using a value of 0.2 [9]. Using this value, a prevalence of the risk factor among controls of 0.4, power of 0.80, and type I error rate of 0.05, we used PASS statistical software v14.0.4 (Released: October 29, 2015; Kaysville, Utah) to determine that a target sample size of 50 cases matched 5:1 with 250 controls would detect a minimum odds ratio (OR) of 2.5.

### Design, enrollment, and data collection

We matched five controls to each case on four variables: sex, age in years at rash onset, district of residence, and urban/rural place of residence. We selected the controls randomly without replacement (i.e., a control could only serve as a control for one case) from the surveillance list of cases.

We developed an abstraction tool to collect relevant information about the study participants. Once trained on the study tool, surveyors conducted from December 2018 to March 2019 1) a review of national case-based surveillance data for measles, 2) a review of hospital medical charts, and 3) a review of family doctor medical records through interviews with family doctors. The interview and data abstraction tool was developed for the purpose of this study (Supplementary File 1). From these data sources, surveyors abstracted relevant data on the selected cases and controls. Variables abstracted included patients’ demographics (age, sex), underlying medical conditions, vaccination status as documented in the family doctor medical record (MCV, pentavalent conjugate vaccine, *Haemophilus influenzae type b* or hexavalent vaccine), healthcare exposures before measles rash onset (including diagnoses), clinical course of measles illness (date of rash onset, signs and symptoms, and complications), and clinical management at the health facility (i.e., vitamin A and other medications received, X-ray results). All cases and controls had to have a known date of rash onset, and date of hospital admission to be included in the study. There were no missing values for these variables. The signs and symptoms that were reported in hospital medical charts were collected systematically as well as any inpatient visit 7 to 21 days prior to rash onset. If a sign was not reported (i.e., conjunctivitis), it was considered not present; if a previous inpatient visit was not reported, it was considered as not present. The preexisting condition, malnutrition, was assessed after the review of family doctor medical records during the interviews with family doctors: if the condition was reported in the medical records, the value was collected as such based on the clinician own evaluation.

### Data analysis

Similar to Lee et al. (2019) methods and investigation in Mongolia [10], we evaluated the following characteristics as potential risk factors for measles death: comorbidities (malnutrition, immune deficiency, anemia); complications of measles infection (encephalitis, seizure, meningitis, pneumonia, diarrhea); receipt of treatment (vitamin A, antibiotics); and potential health care exposure to measles. We defined healthcare-related exposure to measles (HEM) as a history of hospitalization for any cause in the 7 to 21 days before measles rash onset (incubation period) in a confirmed measles case. We created dichotomous variables for HEM for a pneumonia or influenza hospitalization, HEM for other hospitalization (i.e., diarrheal disease, upper respiratory tract infection, urinary tract infection, meningitis, or other), and non-HEM (i.e., no history of hospitalization in the 7–21 days before rash onset) [10].

We analyzed data using RStudio, Inc., Boston, MA [URL http://www.rstudio.com/]. We conducted a univariable analysis by calculating matched ORs and corresponding 95% confidence intervals (95% CI) using conditional logistic regression to compare discordant pairs of matched cases and controls. We also conducted a multivariable analysis by calculating multivariable matched ORs; we initially included all factors used in a univariable model and removed factors until the Akaike information criterion (AIC) was no longer reduced. Multiple independent variables were included based on results from the univariable analysis and the literature [10–13].^1^

This study was determined not to be human subjects’ research by the US Centers for Disease Control and Prevention (CDC) and the Romania National Centre for Communicable Diseases Surveillance and Control, since the primary intent was epidemic disease control activity.

## Results

### Case-control sample characteristics

Of the 300 children included in the study, 231 (77%) had laboratory-confirmed measles, including 92% of the study cases and 74% of the study controls; the remaining 69 (23%) cases and controls were epidemiologically linked to a laboratory-confirmed measles case. There were no differences between case-patients and controls for the matching variables: age at rash onset, sex, or region of residence (Table 1). All Romanian regions were represented in the case-control study except Bucuresti-Ilfov because no fatal measles cases occurred in children aged < 60 months in the municipality. Only 7.0% of all those enrolled (2.0% of the cases and 7.6% of the controls) reportedly received a valid dose of MCV. Among those enrolled, 5.3% were malnourished (12.0% of cases, 4.0% of controls).

**Table 1:** Characteristics of fatal measles cases and matched non-fatal measles controls — Romania, 2016–2018

Characteristic	Cases (***n*** = 50) n (%)	Controls (***n*** = 250) n (%)	***p*** value^**a**^
**Case classification (yes/no)**			
Lab-confirmed	46 (92%)	185 (74%)	
Epidemiologically linked	4 (8%)	65 (26%)	
**Demographic Factors**			
**Age at Rash Onset**^c^ **(yes/no)**			
< 1 year	33 (66.0)	165 (66.0)	
1 year	10 (20.0)	50 (20.0)	
2 years	4 (8.0)	20 (8.0)	
3 years	2 (4.0)	10 (4.0)	
4 years	1 (2.0)	5 (2.0)	
**Sex**^c^ **(yes/no)**			
Male	23 (46.0)	115 (46.0)	
Female	27 (54.0)	135 (54.0)	
**Region of residence**^c^ **(yes/no)**			
Center	2 (4.0)	10 (4.0)	
North-East	15 (30.0)	75 (30.0)	
North-West	3 (6.0)	15 (6.0)	
South	3 (6.0)	15 (6.0)	
South-East	7 (14.0)	35 (14.0)	
South-West	6 (12.0)	30 (12.0)	
West	14 (28.0)	70 (28.0)	
**Medical and Vaccination History**			
**Received ≥ 1 Dose of Vaccine (yes/no)**			
MCV	6 (12.0)	43 (17.2)	0.288
Valid MCV^d^	1 (2.0)	19 (7.6)	0.326^b^
Hib/Hexavalent	20 (40.0)	145 (58.0)	0.124
PCV	5 (10.0)	35 (14.0)	0.547
Influenza	1 (2.0)	1 (0.4)	0.290
**Underlying medical conditions (yes/no)**			
Premature birth	7 (14.0)	19 (7.6)	0.105
Malnutrition	6 (12.0)	10 (4.0)	0.027
Neurologic illness	3 (6.0)	4 (1.6)	0.084
Immune deficiency	1 (2.0)	9 (3.6)	0.569
Anemia	4 (8.0)	19 (7.6)	0.904
Diarrheal illness	0 (0.0)	20 (8.0)	0.997
**Healthcare Exposures Before Measles Rash Onset**			
**Admitted to hospital 7–21 days before rash onset (yes/no)**	18 (36.0)	65 (26.0)	0.134
**Type of hospital**			
District and Local hospitals	3 (6.0)	29 (11.6)	REF
Emergency hospital	10 (20.0)	18 (7.2)	0.123
Specialty hospital	5 (10.0)	11 (4.4)	0.999
**Diagnoses during pre-measles hospitalization (yes/no)**			
Pneumonia	11 (22.0)	23 (9.2)	0.110
Bronchitis	5 (10.0)	6 (2.4)	0.172
Laryngitis	2 (4.0)	3 (1.2)	0.289
Diarrhea	0 (0.0)	12 (4.8)	0.998

^a^Exact conditional logistic regression tests that the distribution of the study participants was different between the case and control groups, excluding “Unknown” categories

^b^ Fisher’s exact test

^c^Matching variable

^d^Received > = 9 months of age and > = 28 days before rash onset

### Pre-measles exposures and clinical course of measles

Table 1 shows the results of healthcare exposures before rash onset. Thirty-six percent of case-patients and 26% of controls were admitted to a hospital during the measles incubation period (i.e., 7 to 21 days before measles rash onset) suggesting healthcare exposure to measles. The case-patients (20%) were more likely than the controls (7%) to be admitted to an emergency hospital during their incubation period. Multiple diagnoses could be reported as their reason for hospitalization before rash onset. Pneumonia was the most commonly reported diagnosis during these hospitalizations—present to a larger extent in measles fatal cases (22%) than in measles non-fatal controls (9%). There was no statistically significant difference between the cases and controls regarding healthcare exposures before measles rash onset (i.e., hospital admission, type of hospital, diagnoses).

The mean duration between measles rash onset and hospitalization for measles was 2 days (range 0–147) and did not differ significantly between the cases and controls. Findings from the clinical course and case management after rash onset are shown in Table 2. Fifty percent of the case-patients and 58% of the controls were hospitalized less than 2 days after measles rash. The type of hospital where most measles cases were admitted after measles rash onset was a specialty hospital (42%), such as infectious disease hospitals and pediatrics hospitals. The fatal cases were statistically more likely to have been admitted to a specialty (*p* < 0.05) or emergency (*p* < 0.05) hospital than to a district, a local, or a clinical hospital. There was no statistically significant difference between the cases and controls regarding the administration of Vitamin A or of antibiotics (Table 1). However, it is notable that only 10% of the case-patients and 4% of the controls received any Vitamin A supplementation to prevent measles complications once the infection was identified.

**Table 2:** Clinical course and case management among fatal measles cases and non-fatal measles controls — Romania, 2016–2018

Variable	Cases (n = 50)n (%)	Controls (n = 250)n (%)	***p*** value^**a**^
**Clinical course & case management**			
**Days to hospitalization after rash onset**			
0 day	18 (36.0)	80 (32.0)	REF
1 day	7 (14.0)	66 (26.4)	0.116
> 1 day	16 (32.0)	60 (24.0)	0.452
**Type of hospital admitted**			
District, Local and Clinical hospitals	9 (18.0)	80 (32.0)	REF
Emergency hospital	8 (16.0)	25 (10.0)	0.015
Specialty hospital	24 (48.0)	101 (40.4)	0.016
**Treatment received (yes/no)**			
Vitamin A (any)	5 (10.0)	10 (4.0)	0.076
Antibiotics (any)	47 (94.0)	225 (90.0)	0.997
**Respiratory complications (yes/no)**			
Bronchitis	18 (36.0)	23 (9.2)	0.000
Laryngitis	3 (6.0)	27 (10.8)	0.289
Pneumonia	45 (90.0)	180 (72.0)	0.002
Acute respiratory distress syndrome	27 (54.0)	16 (6.4)	0.000
Influenza	1 (2.0)	0 (0.0)	0.997
**Chest radiograph findings (yes/no)**			
Bilateral consolidation	13 (26.0)	4 (1.6)	0.000
Interstitial pattern	14 (28.0)	75 (30.0)	0.899
None performed	18 (36.0)	124 (49.6)	0.093
**Neurologic complications (yes/no)**			
Encephalitis	3 (6.0)	3 (1.2)	0.051
Meningitis	2 (4.0)	0 (0.0)	0.998
Seizure	4 (8.0)	4 (1.6)	0.028
**Other complications (yes/no)**			
Diarrhea	4 (8.0)	61 (24.4)	0.009

^a^Exact conditional logistic regression tests that the distribution of the study participants was different between the case and control groups, excluding “Unknown” categories

A higher proportion of fatal cases (90%) were diagnosed with pneumonia following measles infection (*p* < 0.05) compared with the controls (72%) (Table 2). Influenza was identified in one fatal case. Fatal cases were statistically more likely to have respiratory complications of measles such as bronchitis, pneumonia, or acute respiratory distress syndrome (*p* < 0.05). Furthermore, fatal cases (26%) were more likely to have a bilateral consolidation on their chest radiograph than the measles controls (2%). Additionally, fatal cases were more likely to have neurologic complications than measles controls, including encephalitis and seizure (*p* < 0.05).

### Risks for death from measles

Table 3 shows the results from the univariable and multivariable conditional logistic regression for measles mortality among cases and controls. Malnutrition (OR: 3.4; 95% CI [1.1, 9.9]) and inpatient admission with pneumonia during the incubation period were significantly associated with death (OR: 3.0; 95% CI [1.2, 7.2]). Pneumonia (OR: 7.1; 95% CI [2.0, 24.8]) and neurologic complications of measles were also significantly associated with death (OR: 8.7; 95% CI [2.6, 29.1]). After adjustment for covariates, pneumonia as a complication of measles remained significantly associated with measles-related death (OR: 7.1; 95% CI [1.4, 35.3]). The differences in healthcare exposure to measles, history of malnutrition, and neurologic complications between the fatal cases and non-fatal controls were less pronounced after adjusting for other covariates included in the model.

**Table 3:** Univariable and Multivariable Conditional Logistic Regression for Measles Mortality among fatal measles cases and non-fatal measles controls

Variable	Univariable^a^ OR (95% CI)	Multivariable^c^ OR (95% CI)
**Children Medical and Vaccination History**		
Received ≥1 Dose of Vaccine		
MCV	0.6 (0.2–1.6)	1.0 (0.3–3.6)
Hib/Hexavalent	0.6 (0.3–1.2)	0.5 (0.2–1.2)
Underlying medical conditions		
Malnutrition	**3.4 (1.1–9.9)**	4.3 (0.9–20.4)
Immune deficiency	0.5 (0.1–4.4)	0.7 (0.1–6.5)
Anemia	1.1 (0.3–4.4)	2.9 (0.2–39.8)
**Healthcare Exposures Before Rash Onset**		
Admitted to Hospital 7–21 days Before Rash Onset		
No (non-HEM^b^)	REF	REF
HEM: Pneumonia	**3.0 (1.2–7.2)**	1.3 (0.3–5.7)
HEM: Other Diagnosis	1.2 (0.5–2.9)	0.8 (0.2–2.7)
**Healthcare Exposures After Rash Onset**		
Received Vitamin A (any)	3.2 (0.9–11.5)	3.4 (0.7–15.3)
Pneumonia	**7.1 (2.0–24.8)**	**7.1 (1.4–35.3)**
Neurologic complications	**8.7 (2.6–29.1)**	5.7 (0.7–43.9)

^a^Conditional logistic regression odds ratio (OR) accounting for age, sex, district of residence and urban/rural place of residence

^b^*HEM* Healthcare-related Exposure to Measles

^c^Conditional logistic regression odds ratio (OR) accounting for matching on age, sex, district of residence, urban/rural place of residence and controlling for all other variables in this table

## Discussion

Pneumonia as a complication of measles was an independent statistically significant risk factor for death. In our study, 90% of the cases and 72% of the controls had pneumonia as a complication of measles. These findings support results found in other studies stating that coinfections with other respiratory pathogens have been associated with increased severity of disease [14–17]. In a study from Mongolia, Lee et al. (2019) explain that measles can be a “second hit” for previously ill children and infants with acute respiratory infections such as influenza [10]. Other indications of the severity of infection in cases and controls included neurologic complications, such as encephalitis and seizures. Measles encephalitis is regarded as a rare complication occurring in only approximately 0.1% of measles cases [18]. The key pathogenic mechanism in postinfectious encephalitis and/or myelitis (PIE) is believed to be demyelination mediated by an autoimmune process during the stage of recovery from measles infection, generally within 2 weeks of the rash [18].

The use of Vitamin A among both measles cases and controls in this study was very low. The higher proportion of cases receiving Vitamin A compared to the measles controls suggests that prescription was driven by measles severity on a case-by-case basis. Vitamin A deficiency is a recognized risk factor for severe measles infection and death [16]. Vitamin A deficiency may correlate with lower measles-specific antibody levels and increased morbidity. In a child with malnutrition, vitamin A deficiency can result in little to no hepatic reserves of vitamin A when infected with measles [19, 20]. The WHO recommends vitamin A administration once a day for two consecutive days to children with measles (once daily dosages: 50,000 international unit (IU) to infants less than 6 months old, 100,000 IU to infants aged 6–11 months, and 200,000 IU to children older than 1 year of age) [21]. Our findings suggest a gap between recommendations and practice for hospital management of measles that has been reported in other settings [22]. Adopting WHO recommendations for Vitamin A supplementation is critical to prevent the complications of measles in infected patients.

Our investigation showed that malnutrition was associated with measles death. Previous studies have also found an association between malnutrition and measles morbidity [11–13]. One study found that measles mortality rates were progressively higher among children as the degree of wasting increased [14]. Because measles infection transiently depresses numbers of circulating lymphocytes and impairs delayed cutaneous hypersensitivity, it has been hypothesized that malnutrition may increase the severity of measles by inducing an extra burden on cell-mediated immunity [23]. However, other investigators question the connection between malnutrition and measles, positing that previous studies did not adequately control for other confounding variables, such as crowding and poor access to care [24].

A nationwide MCV campaign with high coverage targeting a wide age range is urgently needed to decrease the burden of measles in Romania and to make progress towards measles elimination. Romania experienced a large nationwide measles outbreak from 2016 through 2018 due to transmission among unvaccinated individuals of all ages. Despite a commitment to reaching elimination as part of the WHO European Regional measles elimination goal by 2020, the incidence of measles in Romania was the highest among the 53 member states in 2016 at 124 cases per million and in 2017 at 464 cases per million, and it was the fifth highest measles incidence within the region in 2018 at 327 per million [4, 5]. The wide age range of cases in the 2016–2018 outbreak indicate weaknesses in the delivery of childhood routine immunization services, as well as measles susceptibility gaps among adults.

Stronger support to the legislative process is recommended to strengthen routine immunization operations and surveillance and outbreak response capacity. Romania faces similar challenges with its immunization program operations, including outbreak response capacity, as do other MICs [25]. The Romanian national health authorities have attempted to reduce the high incidence of measles and improve declining coverage. However, the immunization law developed to improve the operations of the National Immunization Programme, to strengthen outbreak response capacity, and to make immunization mandatory is yet to be enacted.

In this study, one of the 50 measles-related deaths occurred in a patient who had received a valid dose of an MCV. The vaccinated case-patient was a four-year-old who received vaccine at 13 months of age. However, this child had a history of malnutrition, which may have resulted in lowered vaccine effectiveness. During measles infection, the child was diagnosed with several additional conditions, including pneumonia resulting in acute respiratory insufficiency, and anemia. The other 5 case-patients who had been vaccinated had not received a valid dose; they had not received the MCV dose at appropriate age or interval before measles rash onset to count as valid dose. One dose of an MCV is approximately 93% effective against measles, while two doses are about 97% effective [26]. Receiving an MCV before 9 months of age results in a vaccine effectiveness of approximately 51%, which is much lower than the effectiveness at nine and 12 months of age (83 and 93% respectively) [27, 28]. Additionally, a child was admitted 147 days after rash onset; this case may have suffered from long-term respiratory complications such as chronic lung disease. Pneumonia was the reason for his admission as well as purulent conjunctivitis. However, we cannot exclude that retrospective data collection may have affected the quality of the data collected.

Infection prevention and control measures should be implemented in every hospital type to prevent nosocomial measles virus transmission. Measures include rapidly identifying and isolating patients with known or suspected measles, and health care workers adhering to standard and airborne precautions [21, 29]. Ensuring that all healthcare staff are immune to measles prior to employment and hospitalizing only severe measles cases may further aid in decreasing nosocomial transmission in Romania. There is a generally low threshold for hospitalizing mildly ill children in Romania. Measles cases in Romania are routinely hospitalized, even those without severe disease. Approximately one-third of the study participants were hospitalized during their measles incubation period, suggesting that their infections may have been associated with healthcare exposure. Measles outbreaks in hospitals can result in more severe disease and death due to transmission among already ill persons. Such outbreaks in hospitals and other congregate settings can be particularly serious due to the high contact rates and transmission potential, and the ability of the measles virus to remain viable in aerosol suspension for up to 2 h [29, 30]. Susceptible healthcare staff are frequently implicated in the amplification and proliferation of measles outbreaks because of exposure and transmission in their work environment [30–32]. WHO recommends that all healthcare workers should be immune to measles and a proof of vaccination or immunity should be a condition of employment and enrollment in trainings [27]. We observed increased mortality in specialty hospitals (i.e., infectious disease, pediatrics, cancer) which may be due to the fact that patients admitted in specialty hospitals are more likely to have chronic or severe diseases than those admitted into district and local hospitals.

There are limitations to this study which may influence the interpretation of our results. Because we restricted the case control sample to infants and children < 5 years of age hospitalized with measles infection, we were unable to make inferences about other age groups. The choice of this population age prevented the identification of young age as a risk factor for death. However, our goal was to understand why infants and children under 5 years of age were disproportionately affected by measles-related death. The assessment of respiratory co-infection with other circulating pathogens was limited to influenza as recorded in the medical charts with poor access to laboratory data for confirmation. Although we followed the WHO measles-related death definition [8], post-mortem evaluations were not conducted to ensure that measles deaths were indeed caused by the measles virus and not caused by any other co-circulating pathogens or any other underlying medical conditions. Thirdly, as medical record abstraction was used to obtain data for the study, other determinants of health could not be measured as potential risk factors for mortality (e.g., household income level or access to care). However, cases and controls were matched on district and on urban/rural place of residence, which likely controlled for other unmeasured confounders. Finally, we were unable to directly measure vitamin A deficiency, although malnutrition and anemia were recorded and their association with disease outcome was measured.

## Conclusions

Because of a safe and effective measles vaccine, each death related to measles is a “preventable tragedy that could have been avoided through vaccination” [26]. Our investigation highlighted factors that may aid in future outbreak responses, reduce measles-related deaths, and allow Romania to make progress towards their measles elimination goal. Hospitalizing only severe measles cases, ensuring that all healthcare staff are immune to measles prior to employment, and implementing infection prevention and control practices in every hospital may aid in decreasing nosocomial transmission in Romania. National health authorities should consider adopting and implementing evidence-based strategies to increase MCV routine immunization coverage and should conduct supplemental immunization activities to address nationwide measles immunity gaps. Ensuring that infants are vaccinated for diseases that result in respiratory illnesses may also help reduce measles-related mortality in Romania. Finally, adopting WHO recommendations for Vitamin A supplementation is critical to prevent the complications of measles in infected patients.

### Disclaimer

The findings and conclusions in this report are those of the authors and do not necessarily represent the official position of the US Centers for Disease Control and Prevention.

The authors alone are responsible for the views expressed in this article and they do not necessarily represent the views, decisions or policies of the institutions with which they are affiliated.

## Supplementary Information

**Additional file 1: Supplementary File 1.** Data abstraction tool, measles case-control study in Romania, 2016–2018.

## Data Availability

The Romanian national measles surveillance databases are available publicly (http://www.cnscbt.ro/index.php/informari-saptamanale/rujeola-1/). Hospital and family doctor medical records are private. The datasets used and/or analyzed during the current study are available from the corresponding author on reasonable request.

## Abbreviations

AIC: Akaike information criterion
CDC: Centers for Disease Control and Prevention
CFR: Case fatality ratio
CI: Confidence interval
HEM: Healthcare-related exposure to measles
IgG: Immunoglobulin G
IgM: Immunoglobulin M
IU: International unit
MCV: Measles-containing vaccine
MCV1: First dose of measles-containing vaccine
MCV2: Second dose of measles-containing vaccine
MIC: Middle-income country
MOH: Ministry of Health
OR: Odds ratio
PIE: Postinfectious encephalitis and/or myelitis
RNA: Ribonucleic acid
RT-PCR: Reverse transcription polymerase chain reaction
WHO: World Health Organization

## Footnotes

1 Prior studies had identified neurological complications, acute malnutrition, certain hospital settings, residing in critically insecure or remote areas, being unvaccinated against measles, not receiving vitamin A during the illness, and being the first child in the household to have measles as independent risk factors for mortality.
