# Supplementary material for: Risk factors for measles deaths among children during a Nationwide measles outbreak – Romania, 2016–2018
Source: BMC Infect Dis. 2021 Mar 19;21:279. doi: 10.1186/s12879-021-05966-3 (PMC7976682; doi:10.1186/s12879-021-05966-3)
Supplement: Supplementary file 1 — Additional file 1: Supplementary File 1. Data abstraction tool, measles case-control study in Romania, 2016–2018. [file 12879_2021_5966_MOESM1_ESM.docx]

**Supplementary File 1: Abstraction tool**

**IDENTIFICATION**

| **Q** | **Label** | | **Type** | **Coding** |
| --- | --- | --- | --- | --- |
| **A. CASE DETAILS** | | | | |
| 1 | Abstraction ID | | Numeric | Numeric |
| 2 | Name of Abstractor | | Categorical | List of abstractor name |
| 3 | Unique Identifier | | 12-digit character | XX-XXXXXXXXXX  (Match ID-National Numeric ID Number) |
| 4 | Date of Birth | | Date | DD-MM-YYYY  99= Unknown |
| 5 | Date of Rash Onset | | Date | DD-MM-YYYY  99= Unknown |
| 6 | Case Status | | Binary | 0 = Control  1 = Case (died) |
| 7 | Calculate incubation period (7-21 days before rash) – FOR ABSTRACTOR USE ONLY | | Date range | DD-MM-YYYY to DD-MM-YYYY |
| 8 | Age (in years) | | Numeric | Numeric, in years |
| 9 | Sex | | Binary | 0 = Male  1 = Female |
| 10 | District of residence (permanent residence) | | Categorical | Standardized list |
| 11 | Residence of case within the district | | Categorical | 0= Urban  1= Rural |
| 12 | Other district visited during the incubation period (non-permanent residence) | | Categorical | Standardized list  99= NA |
| **B. CASE CLASSIFICATION** | | | | |
| 13 | | Case classification | Categorical | 0= Laboratory confirmed  1= Epi-linked |
| 14 | | If laboratory confirmed, specify the methods | Categorical | 0= IgM Serology  1= PCR  2= Other  3= N/A (epi-linked)  99= Unknown |
| 15 | | If ‘Other’, specify | Character | Free Text |

**FAMILY DOCTOR**

| **Q** | **Label** | **Type** | **Coding** |
| --- | --- | --- | --- |
| **FAMILY DOCTOR** | | | |
| 16 | Patient registered with a family doctor | Categorical | 0 = No  1 = Yes  99= Unknown |
| 17 | Name of the family doctor | Character | Free text |
| 18 | District of family doctor | Categorical | Standardized list |
| 19 | Person interviewed | Categorical | 0= Registered family doctor (as mentioned on the header)  1= Nurse  2= Office manager  3= Other |
| 20 | If ‘Other’, please specify | Character | Free Text |
| **B. BIRTH DATA** | | | |
| 21 | Documented premature | Categorical | 0 = No  1 = Yes  99 = Unknown |
| **C. CHILD HISTORY** | | | |
| 22 | Any routine Vitamin A dose received | Categorical | 0 = None  1 = At least one  99 = Unknown/NA |
| 23 | If yes, number of doses | Categorical | 0 = None  1 = One  2 = Two  3 = Three  99 = Unknown/NA |
| **D. MEDICAL HISTORY** | | | |
| 24 | Underlying conditions  CHECK ALL THAT APPLY | Categorical | 0 = Cancer  1 = HIV  2 = Immune deficiency  3 = Birth defect  4 = Rickets  5 = Anemia  6 = Tuberculosis  7 = Twin  8 = Malnutrition  9 = Respiratory illness  10 = Diarrheal illness  11 = Urinary illness  12 = Neurologic illness  13 = No other preceding illness  14 = Other  99 = Unknown |
| 25 | If ‘Other’, specify | Character | Free Text |
| **D. MEASLES HISTORY** | | | |
| 26 | Received any measles vaccine | Categorical | 0 = None  1 = At least one  2 = Not eligible < 9 mo  99 = Unknown |
| 27 | Total number of doses of MCV received | Categorical | 0 = None  1 = One  2 = Two  3 = Three  99 = Unknown |
| 28 | If received 0 dose, reason  CHECK ALL THAT APPLY | Categorical | 0 = Neomycin allergy  1 = Age Ineligible (< 9 mo)  2 = Severe Respiratory failure  3= Vaccine not available at the health facility (ie, national vaccine stock-out)  4= Immunocompromised  5= Parental refusal  6= Other  7= Unknown/NA |
| 29 | If ‘Immunocompromised’ or ‘Other’, specify | Character | Free Text |
| **E. OTHER VACCINATION HISTORY** | | | |
| 30 | Any Hib-containing vaccine received? | Categorical | 0 = None  1 = At least one  99 = Unknown/NA |
| 31 | If yes, number of doses | Categorical | 0 = None  1 = One  2 = Two  3 = Three  99 = Unknown/NA |
| 32 | Any pneumococcal conjugate vaccine (PCV) received? | Categorical | 0 = None  1 = At least one  99 = Unknown/NA |
| 33 | If yes, number of doses | Categorical | 0 = None  1 = One  2 = Two  3 = Three  99 = Unknown/NA |
| 34 | Received influenza vaccination ever? | Categorical | 0 = No  1 = Yes  99 = Unknown/NA |
| 35 | If yes, date of most recent dose received | Date | DD-MM-YYYY  99 = Unknown/NA |

**INPATIENT VISITS BEFORE CURRENT HOSPITALIZATION**

|  | **Variable** | **Type** | **Coding** | |
| --- | --- | --- | --- | --- |
| **H. INPATIENT VISIT BEFORE CURRENT HOSPITALISATION (either from hospital or family doctor records)** | | | | |
| 36 | Been to a hospital 7 to 21 days prior to rash onset | Categorical | | 0 = No  1 = Yes  99 = Unknown |
| 37 | District of hospital | Categorical | | Standardized list |
| 38 | Date of most recent hospital visit before rash onset | Character | | Free Text  99 = Unknown/ NA |
| 39 | Type of hospital | Categorical | | 1= District hospital (spital clinic judetean)  2= Town hospital (spital orasenesc)  3= Specialty hospital (spital clinic de..)  4= Emergency hospital (spital judetean or municipal or clinic de urgenta)  5= Municipal hospital (spital municipal)  6= Long-term hospital for chronic diseases  7= Clinical hospital  8= Institutes and clinical medical centres  9= Health centres |
| 40 | Admitted to ICU at this hospital | Categorical | | 0 = No  1 = Yes  99 = Unknown/NA |
| 41 | Intubated at this hospital | Categorical | | 0 = No  1 = Yes, mechanically ventilated  2 = Yes, hand ventilated  99 = Unknown/NA |
| 42 | Diagnoses in this hospitalization  CHECK ALL THAT APPLY | Categorical | | 0 = Pneumonia  1 = Influenza  2 = Diarrhea  3 = Bronchitis  4 = Laryngitis  5 = Seizure  6 = Meningitis  7 = Urinary Infection  8 = Measles  9 = Other  99=NA |
| 43 | If ‘Other’, specify | Character | | Free Text |
| 44 | Discharge/ Transfer date | Date | | DD-MM-YYYY  99 = Unknown |
| 45 | Developed measles rash or diagnosed with measles during the hospitalization (after admission date)? | Categorical | | 0 = No  1 = Yes  99 = NA |

**CURRENT HOSPITALIZATIONS**

| **HOSPITAL OF THE MEDICAL CHART** | | | | |
| --- | --- | --- | --- | --- |
|  | **Variable** | | **Type** | **Coding** |
| 46 | District of hospital | | Categorical | Standardized list |
| 47 | Type of hospital | | Categorical | 1= District hospital (spital clinic judetean)  2= Town hospital (spital orasenesc)  3= Specialty hospital (spital clinic de..)  4= Emergency hospital (spital judetean or municipal or clinic de urgenta)  5= Municipal hospital (spital municipal)  6= Long-term hospital for chronic diseases  7= Clinical hospital  8= Institutes and clinical medical centres  9= Health centres |
| 48 | Name of hospital | | Character | Free Text |
| 49 | Admission date | | Date | DD-MM-YYYY  99 = Unknown |
| 50 | Discharge/transfer/death date | | Date | DD-MM-YYYY  99 = Unknown |
| **G. CURRENT HOSPITALISATION (HOSPITAL OF THE MEDICAL CHART)** | | | | |
| 51 | Admitted to ICU at this hospital | Categorical | | 0 = No  1 = Yes  99 = Unknown/NA |
| 52 | Intubated at this hospital | Categorical | | 0 = No  1 = Yes, mechanically ventilated  2 = Yes, hand ventilated  99 = Unknown/NA |
| 53 | Presenting symptoms  CHECK ALL THAT APPLY | Categorical | | 0 = Maculopapular rash  1 = Fever  2 = Conjunctivitis  3 = Coryza/ Rhinorrhea  4 = Cough  5 = Difficulty breathing  6 = Wheezing  7 = Weakness  8 = Diarrhea  9 = Vomiting  10 = Poor feeding  11 = Malnutrition  12 = Other  99 -NA |
| 54 | If ‘Other’, specify | Character | | Free Text |
| **Diagnoses DURING CURRENT HOSPITALISATION - CHECK ALL THAT APPLY** | | | | |
| 55 | Measles | Categorical | | 0 = No  1 = Yes  99 = Unknown |
| 56 | Influenza | Categorical | | 0 = No  1 = Yes  99 = Unknown |
| 57 | Diarrhea | Categorical | | 0 = No  1 = Yes  99 = Unknown |
| 58 | Bronchitis | Categorical | | 0 = No  1 = Yes  99 = Unknown |
| 59 | Laryngitis | Categorical | | 0 = No  1 = Yes  99 = Unknown |
| 60 | Urinary Infection | Categorical | | 0 = No  1 = Yes  99 = Unknown |
| 61 | Other | Categorical | | 0 = No  1 = Yes  99 = Unknown |
| 62 | If ‘Other’, specify | Character | | Free Text |
| 63 | Encephalitis | Categorical | | 0 = No  1 = Yes  99 = Unknown |
| 64 | Meningitis | Categorical | | 0 = No  1 = Yes  99 = Unknown |
| 65 | Seizure | Categorical | | 0 = No  1 = Yes  99 = Unknown |
| 66 | Pneumonia diagnosed (clinically) | Categorical | | 0 = No  1 = Yes  99 = Unknown |
| 67 | X-ray findings  CHECK ALL THAT APPLY | Categorical | | 0 = Bilateral consolidation  1 = Unilateral  2 = Lobar consolidation  3 = Interstitial pattern  4 = Reticular pattern  5 = Bilateral hazy opacities  6 = Other  7 = Normal chest X-ray/ no radiographic pneumonia  8 = No X-ray performed  99= Unknown |
| 68 | If ‘Other’, specify | Character | | Free Text |
| 69 | Acute respiratory distress syndrome diagnosed | Categorical | | 0 = No  1 = Yes  99 = Unknown |
| **Clinical management DURING CURRENT HOSPITALISATION** | | | | |
| 70 | Received any Vitamin A dose after rash onset | Categorical | | 0 = None  1 = At least one  99 = Unknown |
| 71 | If ‘yes’, number of doses | Categorical | | 0 = 0 dose  1 = 1 dose  2 = 2 doses  3 = 3 doses  99 = Unknown |
| 72 | If > 0 dose, dosage of first dose | Categorical | | 0 = 50,000 IU  1 = 100,000 IU  2 = 200,000 IU  99 = Unknown/ NA |
| 73 | If > 0 dose, dosage of second dose | Categorical | | 0 = 50,000 IU  1 = 100,000 IU  2 = 200,000 IU  99 = Unknown/ NA |
| 74 | Received rehydration therapy | Categorical | | 0 = No  1 = Yes  99 = Unknown |
| 75 | Received antibiotics | Categorical | | 0 = No  1 = Yes  99 = Unknown |
| 76 | Received corticosteroids | Categorical | | 0 = No  1 = Yes  99 = Unknown |
| 77 | Received Veferon/ Interferon | Categorical | | 0 = No  1 = Yes  99 = Unknown |
| 78 | Received Immunoglobulin/ IV Ig | Categorical | | 0 = No  1 = Yes  99 = Unknown |
| **H. CULTURE DATA DURING CURRENT HOSPITALISATION** | | | | |
| 79 | Respiratory viral PCR performed? | Categorical | | 0 = No  1 = Yes  99 = Unknown |
| 80 | If yes, CHECK ALL THAT APPLY | Categorical | | 0 = Blood  1 = Nasopharyngeal  2 = Urine  3 = CSF  4=Coproculture  5=Bronchial/tracheal aspirate  6=Rectal/perianal tamponage  7-Conjunctival  8-Otic secretion  9-Gastric aspiration  99=NA |
| 81 | If ‘Other’, specify | Character | | Free Text |
| 82 | Bacterial culture performed | Categorical | | 0 = No  1 = Yes  99 = Unknown |
| 83 | If yes, sample 1 | Categorical | | 0 = Blood  1 = Nasopharyngeal  2 = Urine  3 = CSF  4=Coproculture  5=Bronchial/tracheal aspirate  6=Rectal/perianal tamponage  7-Conjunctival  8-Otic secretion  9-Gastric aspiration  99=NA |
| 84 | Name of organism 1 | Character | | Free Text |
| 85 | If yes, sample 2 | Categorical | | 0 = Blood  1 = Nasopharyngeal  2 = Urine  3 = CSF  4=Coproculture  5=Bronchial/tracheal aspirate  6=Rectal/perianal tamponage  7-Conjunctival  8-Otic secretion  9-Gastric aspiration  99=NA |
| 86 | Name of organism 2 | Character | | Free Text |
| 87 | Notes | Character | | Free Text |
| **Outcome of CURRENT HOSPITALISATION** | | | | |
| 88 | Transfer to another hospital? | Categorical | | 0 = No  1 = Yes |
| 89 | If ‘yes’, specify the name of the hospital | Character | | Free Text |
| 90 | Discharged home? | Categorical | | 0 = No  1 = Yes |
| 91 | Death? | Categorical | | 0 = No  1 = Yes |
